# Differential modulation of cisplatin efficacy by montelukast sodium and desloratadine in lung cancer

**DOI:** 10.55730/1300-0152.2771

**Published:** 2025-09-13

**Authors:** Seha AKDUMAN, Büşra YÜKSEL, Didem TECİMEL, Ömer Faruk BAYRAK, Didem SEVEN, Fikrettin ŞAHİN

**Affiliations:** 1 Department of Chest Diseases, School of Medicine, Yeditepe University, İstanbul, Turkiye; 2 Department of Genetics and Bioengineering, Faculty of Engineering, Yeditepe University, İstanbul, Turkiye; 3 Department of Medical Genetics, School of Medicine, Yeditepe University, İstanbul, Turkiye

**Keywords:** Desloratadine, montelukast sodium, cisplatin, lung cancer

## Abstract

**Background/objective:**

Despite advances in treatment, achieving effective and durable responses with chemotherapy remains a significant challenge in lung cancer management. This study investigates the effects of montelukast sodium (MLS) and desloratadine (DES), alone and in combination with cisplatin (CIS), on cell viability, apoptosis, cell cycle distribution, and antioxidant gene expression in A549 and DMS114 lung cancer cell lines.

**Materials and methods:**

Cells were treated with CIS, MLS, DES, and their combinations for 24–72 h. Cell viability was assessed via MTS assay; apoptosis and cell cycle progression were analyzed by flow cytometry. The expression of antioxidant-related genes (*GPX4*, *GSR*, *GCLC*) was quantified using qRT-PCR.

**Results:**

MLS and DES reduced cell viability individually in both cell lines in a dose- and time-dependent manner. The combination of CIS and MLS showed near-synergistic effects in A549 cells. The combination significantly enhanced apoptosis, particularly in DMS114 cells. In contrast, CIS combined with DES showed antagonistic interactions in both lines, with no significant increase in apoptosis compared to CIS alone. MLS combined with CIS also enhanced G0/G1 phase arrest, while the combination of DES and CIS had no additive effect on the cell cycle. DES alone or with CIS significantly upregulated *GPX4* and *GCLC*, suggesting activation of antioxidant defense mechanisms. Meanwhile, MLS alone or combined with CIS led to a decrease in *GCLC* expression, indicating a possible impairment of redox homeostasis.

**Conclusion:**

MLS enhances CIS-induced cytotoxicity and apoptosis in lung cancer cells and modulates redox gene expression, potentially improving therapeutic efficacy. In contrast, DES may attenuate CIS activity through antioxidant gene upregulation. These findings support the potential of MLS as an effective adjuvant in CIS-based lung cancer treatment. However, the antagonistic effect observed with DES highlights the importance of careful evaluation of candidates for drug repurposing.

## 1. Introduction

Lung cancer ranks as the most prevalent cancer globally and remains the leading cause of cancer-related mortality. The average age at diagnosis is typically between 60 and 70 years. Notably, the incidence and mortality rates are higher in developed countries than in less developed regions, such as Central and South America and most parts of Africa. Tobacco use is the primary risk factor for lung cancer, accounting for approximately 80% of all cases (Kratzer et al., 2024). Lung cancer is classified into 2 subgroups histopathologically as small-cell and nonsmall-cell lung cancer (SCLC and NSCLC, respectively). SCLC accounts for approximately 15% of all lung cancers. SCLC cells are distinguished by their rapid proliferative capacity and high malignant potential, making SCLC one of the most aggressive subtypes of lung cancer. SCLC is a highly aggressive cancer with a low 5-year survival rate (<7%) (Meriggi, 2024). NSCLC represents the majority of lung cancer cases diagnosed each year, encompassing several distinct subtypes. Adenocarcinoma is the most common among these, comprising approximately 50% of all NSCLC cases. Squamous cell carcinoma follows as the second most prevalent subtype, accounting for 20–30% of cases. These two subtypes underscore the diverse pathological landscape of NSCLC (Hendriks et al., 2024). The common metastatic organs for lung cancer include the lymph nodes, brain, bones, and adrenal glands (Wei et al., 2024). The first-line treatment of lung cancer is chemotherapy, including cisplatin (CIS) and etoposide, immunotherapies, and targeted therapies. Despite the availability of various treatment modalities, the 5-year survival rate for lung cancer remains alarmingly low, ranging from 10% to 20% (Bi et al., 2024). This underscores the critical and urgent need for advancements in lung cancer treatment strategies.

Drug repurposing involves investigating existing medications originally developed for other diseases to treat cancer, offering significant benefits such as faster development due to established safety profiles, cost effectiveness by reducing the need for extensive early stage testing, and the potential to target diverse molecular pathways relevant to cancer. This approach can also improve patient access by providing more affordable treatments. Repurposed drugs may be combined with existing therapies to enhance efficacy and reduce the risk of drug resistance. Drug repurposing presents a promising avenue for expanding cancer treatment options and addressing the global need for effective therapies (Trybus and Trybus, 2024). In the fight against cancer, well-known antihistamines such as desloratadine (DES) and montelukast sodium (MLS) have shown significant potential (Faustino-Rocha et al., 2017). DES, a second-generation antihistamine, and MLS, a leukotriene receptor antagonist, are primarily used to treat allergies and asthma. Emerging research suggests they may also influence cancer progression (Hu et al., 2023). These drugs have been studied for their ability to inhibit tumor growth, metastasis, and inflammation, potentially offering new therapeutic avenues for cancer treatment (Hamid and Hamidi, 2024). Their low cost, established safety profiles, and accessibility make them attractive candidates for repurposing in oncology, particularly in combination with other cancer therapies. DES was found to be effective in all immunogenic cancer types, including stomach, pancreas, colon/rectum, breast, lung, kidney, bladder, prostate, Hodgkins lymphoma, and melanoma. However, it was ineffective in all nonimmunogenic cancers, such as non-Hodgkins lymphoma, brain/central nervous system cancers, thyroid, liver, uterus, and ovarian cancers (Fritz et al., 2021).

This study aimed to investigate the effects of 2 prevalent antihistamines, DES and MLS, along with their combination with CIS, in lung cancer to elucidate their potential impact on cancer treatment.

## 2. Materials and methods

### 2.1. Cell culture and cell culture conditions

DMS114 (catalog number: CRL-2066, SCLC cell line) and A549 (catalog number: CCL-185, NSCLC cell line) were purchased from American Type Culture Collection (ATCC, Rockville, MD, USA). The DMS114 cell line was cultured in Roswell Park Memorial Institute medium (RPMI, catalog number: 11875093, Invitrogen, Gibco, UK). The A549 cell line was cultured in Dulbeccos Modified Eagles Medium (DMEM, catalog number: 41966-029, Invitrogen, Gibco, UK). Each medium was supplemented with 1% penicillin/streptomycin/amphotericin (PSA, Invitrogen, Gibco, UK) and 10% fetal bovine serum (FBS, catalog number: 10500-064, Invitrogen, Gibco, UK). Cells were maintained at 37 C and 5% CO_2_ in a humidified incubator.

### 2.2. Cell viability assay

The effect of MLS (Pharmachem, New Jersey, USA) and DES (Morepen Lab, Parwano, India) on the viability of DMS114 and A549 cells was tested. Stock solutions of 1 mM MLS and 1 mM DES were dissolved in RPMI for DMS114 and DMEM for A549, containing DMSO in a 1:1000 ratio. CIS was obtained from Koçak Farma (Türkiye) (50 mg/100 mL IV infusion concentrate solution). Stock solution was provided in 0.9% NaCl and further diluted in complete culture medium immediately before use to reach the indicated final concentrations. DES and MLS were dissolved in DMSO, ensuring that the final DMSO concentration did not exceed 0.1%. A vehicle control group (0.1% DMSO-treated cells) was included in all experiments to ensure that observed cytotoxicity was not attributable to the solvent.

DMS114 and A549 cells were cultured in 96-well plates at 5000 cells/well and 2500 cells/well, respectively. The following day, cells were treated with MLS and DES alone (doses ranging from 200 μM to 3.125 μM), CIS/MLS combinations (doses ranging from 100 μM/50 μM to 1.5625 μM/50 μM), or CIS/DES combinations (doses ranging from 100 μM/25 μM to 1.5625 μM/25 μM). To see the additive, synergistic, and/or antagonistic effect in the combination treatments in both cell lines (DMS114 and A549), the fixed dose of MLS or DES was chosen as the dose at which the cells were viable, and CIS was applied in the dose range. The combination index (CI) was determined by the Chou–Talalay theorem (Chou, 2010). After cells were treated with different compound concentrations for 24, 48, and 72 h, cell viability was assessed by MTS assay (catalog number: G3582, CellTiter96 Aqueous One Solution; Promega, Southampton, UK) according to the manufacturers instructions. Briefly, MTS solution (PBS solution included 10% MTS and 4.5 g/L d-glucose solution) was added, followed by 60 min incubation at 37 C. Then, their absorbance was measured at 490 nm using an ELISA plate reader (Biotek, Winooski, VT, USA). IC50 values were calculated using GraphPad Prism software.

### 2.3. Annexin V assay

An Annexin V apoptosis kit (Santa Cruz Biotechnology, Dallas, TX, USA) was used to detect the percentage of cell apoptosis. A549 and DMS114 cells were seeded into T25 flasks at a density of 110,000 cells/mL and 350,000 cells/mL, respectively. The following day, media was aspirated, and cells were treated with 5 μM of CIS (CIS5), 50 μM of MLS (MLS50), 25 μM of DES (DES25), and their combinations. In the A549 cell line, the combination doses were 4.8 μM of CIS + MLS50 and CIS5 + DES25. In the DMS114 cell line, the combination doses were 2.6 μM of CIS + MLS50 and 3.6 μM of CIS + DES25. After 72 h of treatment, the Annexin V assay was performed according to the manufacturers protocol. Briefly, cells were harvested and washed with ice-cold PBS. Then they were resuspended in Annexin V binding buffer and separated into 4 groups (Annexin V, propidium iodide (PI), Annexin V + PI, and negative control (NC)). Cells were incubated for 15 min at room temperature for Annexin V and PI staining. Data was analyzed with FACSCalibur (BD Biosciences, Franklin Lakes, NJ, USA) flow cytometry.

### 2.4. Cell cycle analysis

A549 and DMS114 cells were seeded into T25 flasks at a density of 110,000 cells/mL and 350,000 cells/mL, respectively. After 24 h, the cells were treated with CIS5, MLS50, and DES25, and further incubated for 72 h at 37 C. The cells were harvested and washed with PBS and fixed with 70% ice-cold ethanol for at least 2 h at −20 C. Cell pellets were permeabilized with 0.1 % NP-40 (catalog number: 85124, Thermo Fisher Scientific, Waltham, MA, USA) and incubated with 20 μg/mL RNase (catalog number: EN0531, Thermo Fisher Scientific, Lithuania) at room temperature for 30 min. Finally, cells were stained with PI and immediately analyzed with a 488 nm single laser emitting device within 15 min.

### 2.5. RNA extraction and cDNA synthesis

Total RNA was isolated using the TRIzol Reagent (Invitrogen, Thermo Fisher Scientific, USA). RNA concentration and purity were assessed spectrophotometrically using a NanoDrop instrument (Thermo Fisher Scientific). Complementary DNA (cDNA) was synthesized from 1000 ng of total RNA using the High-Capacity cDNA Reverse Transcription Kit (Applied Biosystems, Thermo Fisher Scientific, USA), following the manufacturers instructions.

### 2.6. Gene expression analysis

Quantitative real-time PCR (qRT-PCR) was performed using the StepOnePlus Real-Time PCR System (Applied Biosystems, Foster City, CA, USA). TaqMan gene expression assays were used to quantify *GPX4*, *GSR*, and *GCLC* mRNA expression levels. Each 10 μL PCR reaction mixture contained 0.5 μL TaqMan gene expression assay, 5 μL TaqMan universal PCR master mix (Applied Biosystems, Rotkreuz, Switzerland), 50 ng of cDNA, and 2.5 μL PCR-grade water. Amplification was carried out under the following cycling conditions: initial denaturation at 95 C for 10 min, followed by 40 cycles of 95 C for 15 s and 60 C for 1 min. Gene expression was normalized to the endogenous reference gene *ACTB*. The relative expression levels were calculated using the 2^−ΔΔCt^ method. All reactions were performed in technical replicates to ensure reproducibility.

### 2.7. Statistical analysis

All data are shown as mean (SD) values. Statistical analysis was performed with a 2-way ANOVA, and graphs were drawn using GraphPad Prism 5 software. Statistical significance was determined at ns: nonsignificant, *p < 0.05, **p < 0.01, ***p < 0.001, and ****p < 0.0001.

## 3. Result

### 3.1. The effects of MLS and DES (alone and in combination with CIS) on lung cancer cell viability

**Figure 1:**
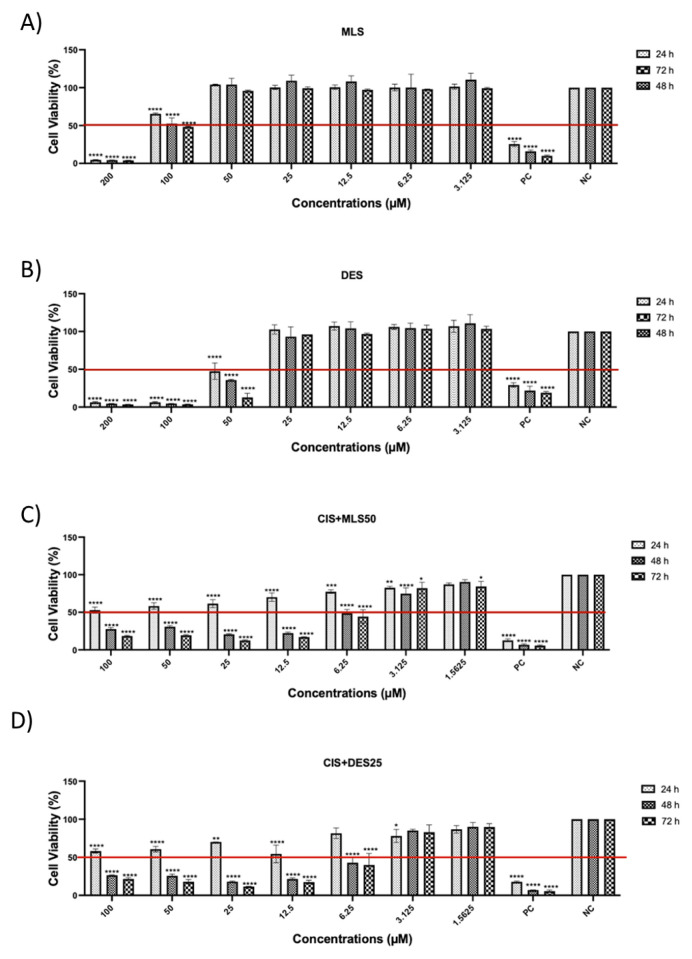
The cell viability of A549 cancer cell line at 24, 48, and 72 h after treatment with (A) MLS, (B) DES, (C) a combination of CIS and MLS, and (D) a combination of CIS and DES. Cell viability was determined by MTS assay and data are expressed as mean and SD (n = 3). Statistical analysis was performed using 2-way ANOVA followed by Dunnetts multiple comparisons test, comparing each treatment concentration with the NC at the same time point. ****p < 0.0001 vs. NC, ns: not significant.

**Figure 2:**
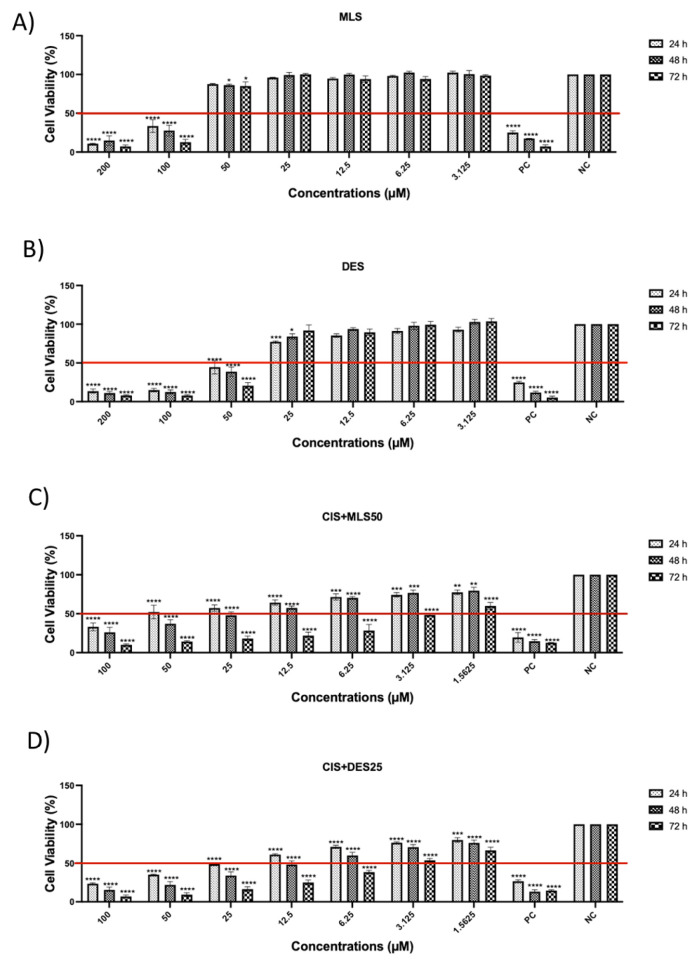
The cell viability of DMS114 cancer cell line at 24, 48, and 72 h after treatment with (A) MLS, (B) DES, (C) a combination of CIS and MLS, and (D) a combination of CIS with DES. Cell viability was determined by MTS assay and data are expressed as mean and SD (n = 3). Statistical analysis was performed using 2-way ANOVA followed by Dunnetts multiple comparisons test, comparing each treatment concentration with the NC at the same time point. ****p < 0.0001 vs. NC, ns: not significant.

This study investigated the effects of CIS alone and in combination with MLS or DES on the viability of A549 and DMS114 lung cancer cell lines. Cell viability was assessed at 24, 48, and 72 h using the MTS assay (Figures 1 and 2). As reported in our previous study (Deniz et al., 2025), the IC50 values of CIS were 10 μM for A549 cells and 5 μM for DMS114 cells. In the present study, IC50 values were additionally determined for MLS, DES, and their respective combinations with CIS at the 72-hour time point (Table). In the combination experiments, CIS was applied at graded concentrations, while MLS and DES were used at fixed concentrations of 50 μM and 25 μM, respectively. Treatment with MLS or DES alone had dose- and time-dependent effects on cell viability. At higher concentrations (≥100 μM), both agents markedly reduced viability in A549 and DMS114 cells. However, at lower concentrations, no significant cytotoxicity was observed. and in some conditions, viability even increased compared with control cells. This highlights the importance of considering dose and time dependency when interpreting the effects of these agents.

When combined with CIS, DES had an antagonistic effect in both A549 and DMS114 cells, as shown by Chou–Talalay analysis, suggesting that DES may interfere with CIS-induced cytotoxicity. In contrast, the combination of CIS with MLS resulted in a CI of 0.99 in A549 cells, indicating a near-synergistic interaction, whereas in DMS114 cells, the effect was less pronounced.

### 3.2. The effects of MLS and DES (alone and in combination with CIS) on apoptosis and cell cycle distribution in lung cancer

**Figure 3:**
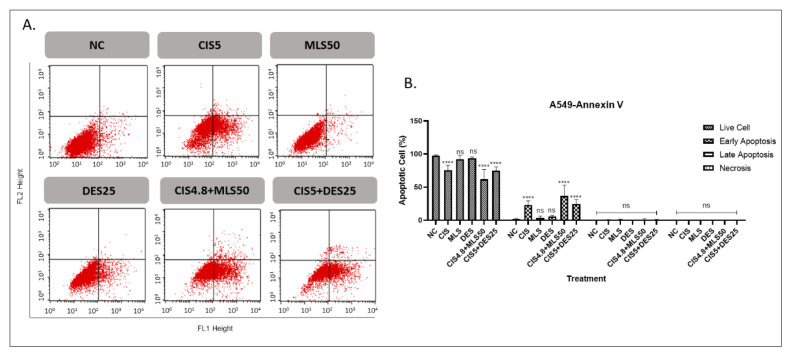
Flow cytometry analysis of apoptosis in A549 cells at 72 h using Annexin V-FITC/PI staining. (A) Representative cytogram: the lower left quadrant indicates live cells (Annexin V^−^/PI^−^), the lower right quadrant represents early apoptotic cells (Annexin V^+^/PI^−^), the upper right quadrant shows late apoptotic cells (Annexin V^+^/PI^+^), and the upper left quadrant indicates necrotic cells (Annexin V^−^/PI^+^). (B) Quantification of apoptotic cells compared to the negative control. Treatments included CIS5, MLS50, DES25, and their combinations. Statistical significance: ns = not significant, *p < 0.05, **p < 0.01, ***p < 0.001, and ****p < 0.0001.

**Figure 4:**
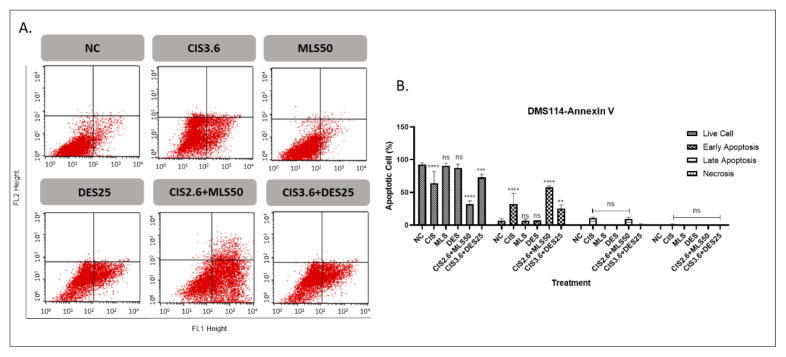
Flow cytometry analysis of apoptosis in DMS114 cells at 72 h using Annexin V-FITC/PI staining. (A) Representative cytogram: the lower left quadrant indicates live cells (Annexin V^−^/PI^−^), the lower right quadrant represents early apoptotic cells (Annexin V^+^/PI^−^), the upper right quadrant shows late apoptotic cells (Annexin V^+^/PI^+^), and the upper left quadrant corresponds to necrotic cells (Annexin V^−^/PI^+^). (B) Quantification of apoptotic cells relative to the negative control. Treatments included CIS5, MLS50, DES25, and their combinations. Statistical significance: ns = not significant, *p < 0.05, **p < 0.01, ***p < 0.001, and ****p < 0.0001.

The proapoptotic effects of individual agents (CIS, MLS, and DES), and their combinations were evaluated in A549 and DMS114 lung cancer cell lines (Figures 3 and 4, respectively). Monotherapy with MLS or DES did not induce significant apoptosis in either cell line. In contrast, CIS alone markedly increased early apoptotic cell populations, reaching 23.2% in A549 cells and 32.3% in DMS114 cells. Combining CIS with MLS notably elevated early apoptosis to 36.7% in A549 and 57.7% in DMS114 cells, representing a significant enhancement over CIS. The combination of CIS and DES also increased early apoptosis compared to the untreated control, the apoptotic response did not significantly differ from that observed with CIS alone. These results suggest that MLS may potentiate CIS-induced apoptosis, particularly in DMS114 cells, whereas DES does not appear to enhance the proapoptotic effect of CIS.

**Figure 5:**
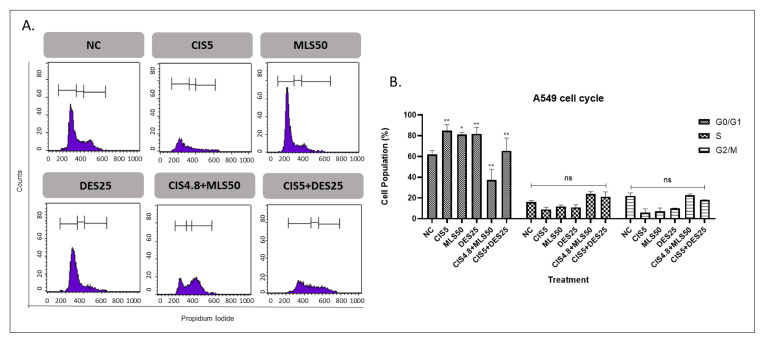
Cell cycle distribution in A549 cells at 72 h. (A) Representative histogram showing the distribution of cell cycle phases, with the initial, middle, and final peaks corresponding to the G0/G1, S, and G2/M phases, respectively. (B) Quantitative analysis of the percentage of cells in G0/G1, S, and G2/M phases compared to the NC. Treatments included CIS5, MLS50, DES25, and their combinations. Statistical significance: ns = not significant, *p < 0.05, **p < 0.01, ***p < 0.001, and ****p < 0.0001.

**Figure 6:**
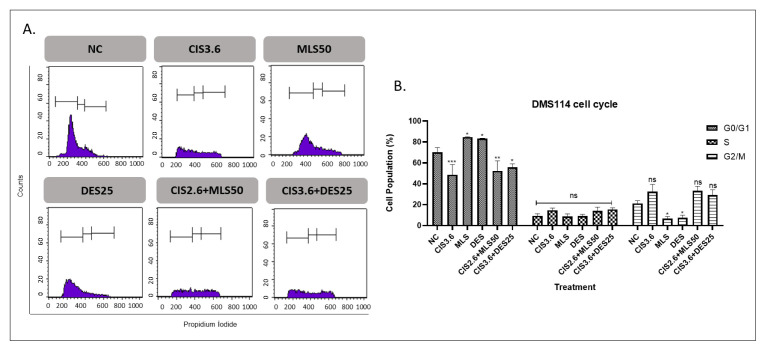
Cell cycle distribution in DMS114 cells at 72 h. (A) Representative histogram depicting cell cycle phases, where the initial, middle, and final peaks correspond to the G0/G1, S, and G2/M phases, respectively. (B) Quantitative comparison of the percentage of cells in G0/G1, S, and G2/M phases relative to the NC. Treatments included CIS5, MLS50, DES25, and their combinations. Statistical significance: ns = not significant, *p < 0.05, **p < 0.01, ***p < 0.001, and ****p < 0.0001.

Single-agent treatments for lung cancer cells with CIS5, MLS50, and DES25 resulted in a statistically significant increase in the G0/G1 phase population compared to the control. MLS and DES alone induced G0/G1 accumulation more compared to CIS treatment alone in DMS114 cells. Notably, combining CIS with either MLS or DES did not further enhance G0/G1 arrest; in fact, the G0/G1 proportion in these combinations was lower than with CIS alone. No significant differences were observed in the S or G2 phases among the combination groups compared with CIS monotherapy (Figures 5 and 6).

### 3.3. Antioxidant gene expression on CIS, MLS, DES, and their combinations on lung cancer cell lines

**Figure 7:**
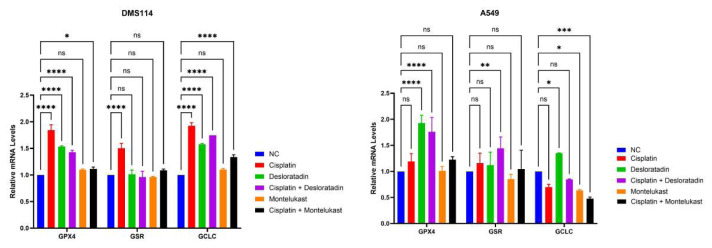
Effect of CIS, MLS, DES, and their combinations on antioxidant gene expression in DMS114 and A549 cell lines. Relative mRNA expression levels of *GPX4*, *GSR*, and *GCLC* were analyzed by qRT-PCR after treatment with CIS (10 μM for A549; CIS5 for DMS114), MLS50, DES25, and their combinations for 72 h. Data are presented as mean ± SEM of 3 independent replicates. ns: nonsignificant, *p < 0.05, **p < 0.01, ***p < 0.001, and ****p < 0.0001.

To further elucidate the molecular mechanisms underlying the effects of CIS, MLS, and DES, the mRNA expression levels of *GPX4*, *GSR*, and *GCLC* were evaluated. These genes are associated with oxidative stress and antioxidant defense. In the DMS114 cell line, CIS alone significantly increased the expression levels of *GPX4*, *GSR*, and *GCLC* compared to the control. Similarly, DES treatment significantly elevated *GPX4* and *GCLC* expression compared to NC, whereas its effect on *GSR* was not statistically significant. The combination of CIS with DES also significantly increased *GPX4* and *GCLC* expression, but had no significant effect on *GSR*. MLS treatment did not significantly alter the expression of *GPX4*, *GSR*, or *GCLC*. Importantly, the combination of CIS and MLS did not significantly change *GPX4*, *GSR*, or *GCLC* mRNA levels, suggesting a distinct molecular response compared to other treatment groups. In the A549 cell line, CIS treatment led to a significant upregulation of *GPX4*; however, *GSR* and *GCLC* expression remained unaltered. DES alone significantly increased *GPX4* expression, while *GSR* expression was modestly but significantly upregulated. The combination of CIS and DES also elevated *GPX4* expression but did not result in significant changes in *GSR* or *GCLC* compared to the control. In contrast, MLS alone or combined with CIS led to a significant decrease in *GCLC* expression compared to control, with no changes in *GPX4* or *GSR* (Figure 7).

## 4. Discussion

This study aimed to investigate the therapeutic potential of MLS and DES, two widely used antiinflammatory and antihistamine agents, in combination with CIS in lung cancer cell lines A549 and DMS114. Our findings show distinct interaction profiles for each agent with CIS, underscoring their differential impacts on cell viability, apoptosis induction, and antioxidant gene expression.

When used as single agents, MLS and DES caused a measurable reduction in cell viability in A549 and DMS114 cell lines. This observation is consistent with earlier reports for MLS, where leukotriene receptor antagonists have shown antiproliferative and proapoptotic effects in lung and other cancer types (Tsai et al., 2017; Marques et al., 2022). For DES, our findings align partially with previous studies in which H1 antihistamines inhibited proliferation in breast and colorectal cancer models (Wen et al., 2021; Oršolić and Jembrek, 2024), although the magnitude of the effect in our lung cancer models was modest. Such differences could be due to variations in histamine receptor expression, drug permeability, or tumor subtype-specific signaling. These drugs, although primarily used for inflammatory or allergic conditions, may therefore possess inherent antiproliferative effects against lung cancer.

The outcomes of combination treatments with CIS showed key differences. The combination of CIS and DES resulted in an antagonistic effect in both cell lines, as indicated by the Chou–Talalay analysis. In light of prior research showing that some H1 antihistamines can interfere with apoptosis signaling, our results suggest that DES may modulate histamine-related pathways or antioxidant defenses in a way that reduces CIS efficacy. Despite a modest reduction in cell viability, the lack of synergism suggests that DES may interfere with the cytotoxic action of CIS, potentially by modulating histamine-related pathways that affect apoptosis or cell survival. This finding aligns with studies that highlight how some H1 antihistamines can have context-dependent or antagonistic interactions with chemotherapeutic agents (Oršolić and Jembrek, 2024). In contrast, combining CIS with MLS had a CI of 0.99 in A549 cells, indicating a near-synergistic or synergistic effect. This is particularly notable, as enhancing the effectiveness of CIS while avoiding additional toxicity remains a major goal in lung cancer therapy. Our results corroborate those of Marques et al. (2022), who showed that MLS, through antagonism of cysteinyl leukotriene receptors, can enhance chemotherapeutic responses by suppressing prosurvival inflammatory signaling and increasing apoptotic sensitivity in cancer cells. Moreover, the observed cell line specificity in synergy, which is stronger in A549 than DMS114, may reflect underlying differences in receptor expression, signaling dynamics, or intrinsic resistance mechanisms between NSCLC and SCLC types. These results emphasize the necessity for tailored strategies when investigating combination therapies and stress the importance of tumor subtype in forecasting response.

The observed G0/G1 phase accumulation following single-agent treatments with CIS, MLS, and DES suggests a modest but statistically significant induction of G1 phase arrest. CIS, a well-characterized DNA crosslinking agent, is known to activate the G1/S checkpoint through p53-dependent and p53-independent pathways in response to DNA damage, thereby halting cell cycle progression to allow for repair or trigger apoptosis (Siddik, 2003; Kelland, 2007). The significant G0/G1 arrest observed with CIS in our study aligns well with its established mechanism of action.

MLS and DES monotherapies also caused modest G0/G1 accumulation, reflecting their intrinsic antiproliferative effects. Interestingly, combining CIS with either MLS or DES did not further enhance G0/G1 arrest; in fact, the G0/G1 fraction was reduced compared with CIS alone. For the combination of CIS and MLS, this attenuation is likely attributable to a shift from cell cycle arrest toward apoptosis, as supported by the marked increase in apoptotic cell populations in both cell lines (Kim et al., 2019). Our data support these findings and suggest that MLS may potentiate the cell cycle arrest effect of CIS, presenting a rationale for further investigating this combination as a potential chemosensitizing strategy.

In contrast, the combination of CIS and DES did not produce significant changes in cell cycle distribution beyond the effect of CIS alone. While DES, an H1 antihistamine, has been shown in some studies to inhibit proliferation and induce apoptosis in cancer cells via oxidative stress and mitochondrial pathways (Wen et al., 2021), its influence on cell cycle regulation appears to be limited or context dependent. The lack of additive effects in this system suggests that, unlike MLS, DES may not synergize with CIS to enhance G1 phase arrest.

In the DMS114 cell line, CIS alone significantly upregulated the expression of all 3 genes (*GPX4*, *GSR*, and *GCLC*), consistent with previous studies indicating that CIS promotes oxidative stress, thereby inducing compensatory antioxidant responses (Jiang et al., 2021). DES also upregulated *GPX4* and *GCLC*, alone and when combined with CIS, while having minimal effect on *GSR*. This pattern suggests that DES may enhance redox buffering capacity through glutathione pathway activation, potentially contributing to the antagonistic interaction observed when combined with CIS. A similar mechanism has been reported in studies where elevated *GPX4* expression has been associated with increased resistance to ferroptosis and reduced chemotherapeutic sensitivity (Friedmann Angeli et al., 2014, 2019; Yang et al., 2014).

By contrast, MLS did not significantly upregulate any of the tested genes in DMS114 cells. More notably, MLS and its combination with CIS led to a significant downregulation of *GCLC* in A549 cells. This finding is mechanistically significant, as *GCLC* catalyzes the rate-limiting step in glutathione synthesis. Reduced *GCLC* expression implies decreased glutathione availability, which may sensitize cells to CIS-induced oxidative stress. This aligns with our earlier viability data where the combination of MLS and CIS had a near-synergistic effect, particularly in A549 cells. Similar glutathione-depleting strategies have been proposed to overcome CIS resistance in various tumors (Traverso et al., 2013).

Interestingly, in A549 cells, both CIS and DES significantly upregulated *GPX4*, while *GSR* and *GCLC* were less affected. The selective increase in *GPX4* suggests the cells were attempting to mitigate lipid peroxidation and ferroptosis. However, this may also represent a resistance mechanism, as high *GPX4* expression is known to protect against oxidative cell death in cancer cells (Stockwell et al., 2017). Collectively, these expression profiles highlight the divergent molecular effects of MLS and DES when combined with CIS. While DES promotes upregulation of antioxidant genes, potentially counteracting the efficacy of CIS, MLS appears to compromise glutathione biosynthesis, particularly via *GCLC* suppression, potentially enhancing CIS-induced cytotoxicity. These contrasting regulatory effects at the transcriptional level may explain the differing patterns of synergy and antagonism observed in the functional assays and underscore the need for molecular profiling when designing combination chemotherapies.

MLS, a cysteinyl leukotriene receptor antagonist primarily used to treat asthma, has recently garnered attention for its antineoplastic properties. Previous studies have suggested that MLS can inhibit cancer cell proliferation, invasion, and survival through modulation of inflammatory pathways and apoptosis (Tsai et al., 2017; Tang et al., 2018). Our research supports these findings and indicates that MLS could improve the effectiveness of CIS by facilitating apoptotic pathways, especially in SCLC models such as DMS114. Additionally, MLS alone did not significantly affect antioxidant gene expression, nor did it in combination with CIS, indicating that its mechanism may be less dependent on oxidative stress pathways and more related to apoptosis induction or other signaling cascades such as NF-κB inhibition or leukotriene receptor modulation.

Conversely, DES, a second-generation antihistamine, reduced cell viability as a single agent but did not enhance the cytotoxic or apoptotic effects of CIS in either cell line. In fact, CI analysis indicated antagonistic interactions in both A549 and DMS114 cells, suggesting that DES may interfere with the mechanism of action of CIS. The influence of DES on antioxidant defense genes could partially explain this antagonism. In our study, DES alone and combined with CIS significantly upregulated *GPX4* and *GCLC* expression in both cell lines. Since *GPX4* is a key enzyme involved in detoxifying lipid peroxides and suppressing ferroptosis (Yang and Stockwell, 2016), its overexpression may contribute to resistance to ferroptosis and increased cancer cell survival. These results align with other studies where upregulation of antioxidant genes correlated with chemotherapy resistance (Cho et al., 2008).

These results indicate that MLS may serve as a potential chemosensitizing agent when combined with CIS, whereas DES might attenuate the therapeutic effectiveness of CIS. This highlights the need for careful molecular evaluation of such combinations before clinical application. Nevertheless, our study has certain limitations: only 2 lung cancer cell lines were investigated, without in vivo validation, and the mechanistic analysis was restricted to a limited set of antioxidant genes. Future studies should address these limitations to better clarify the molecular basis and translational potential of our findings.

## Figures and Tables

**Table:** IC50 values for A549 and DMS114 cell lines after 72 h of treatment.

Treatment	IC50 Values of A549	IC50 Values of DMS114
**Montelukast Sodium (MLS)**	97.04 μM	66.12 μM
**Desloratadine (DES)**	33.93 μM	37.58 μM
**Cisplatin (CIS)/Montelukast Sodium (MLS)**	4.81 μM/50 μM	2.64 μM/50 μM
**Cisplatin (CIS) / Desloratadine (DES)**	5.03 μM/25 μM	3.66 μM/25 μM
